# Statistical analyses on Si microwire solar cells

**DOI:** 10.1016/j.dib.2017.03.030

**Published:** 2017-03-21

**Authors:** Hong-Sik Kim, Joondong Kim

**Affiliations:** Photoelectric and Energy Device Applications Lab (PEDAL) and Department of Electrical Engineering, Incheon National University, 119 Academy Rd. Yeonsu, Incheon 406772, South Korea

**Keywords:** Silicon, Microwire solar cells, Median value, Absolute error

## Abstract

In this data, the statistical analyses of silicon microwire (SiMW) solar cells are presented for the research article entitled “Electrical and optical properties of Si microwire solar cells” (H.-S. Kim, D. B. Patel, H. Kim, M. Patel, K. R. Chauhan, W. Park, and J. Kim) [1]. This article shows the statistical analyses on performances of the various SiMW solar cells. The accuracy of solar cell parameters is discussed on the basis of data sets.

**Specifications Table**

**Table:** 

Subject area	*Electrical Engineering*
More specific subject area	*Solar cells*
Type of data	*Figures*
How data was acquired	*Solar Cell I-V Test System (K3000, McScience)*
Data format	*Raw, analyzed*
Experimental factors	*Each SiMW solar cell was measured 10 times with changing of contact points to observe the variations by contact positing.*
Experimental features	*The error bars of SiMW solar cell parameters were determined.*
Data source location	*Incheon National University, Incheon-406772, Korea*
Data accessibility	*The data are with this article*

**Value of the data**

• The data presents the variations of solar cell parameters with the statistical analyses.
• The variations of solar cell performances were measured by changing of contact positions.
• The data is useful to analyze the accurate solar cell performances by error bars.

## 1 Data

**Fig. 1:**
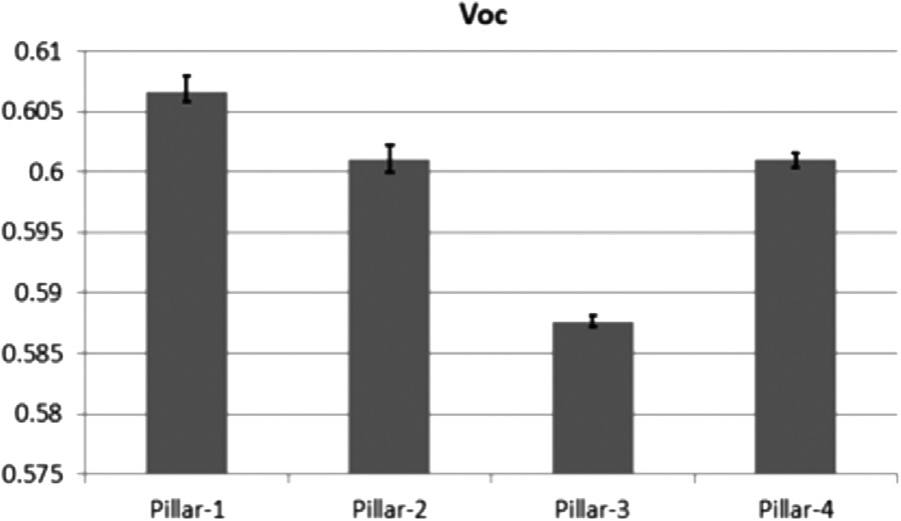
The variation of *V_oc_*. Each bar represents the median value of each solar cell. The remarked line denotes the absolute error range.

**Fig. 2:**
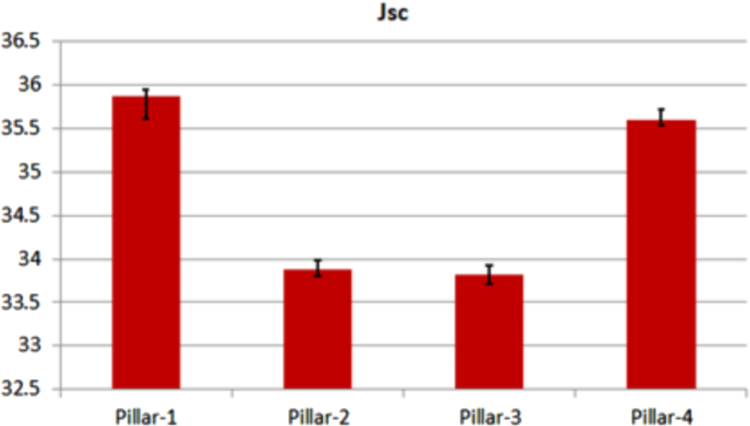
The variation of *J_sc_*. Each bar represents the median value of each solar cell. The remarked line denotes the absolute error range.

**Fig. 3:**
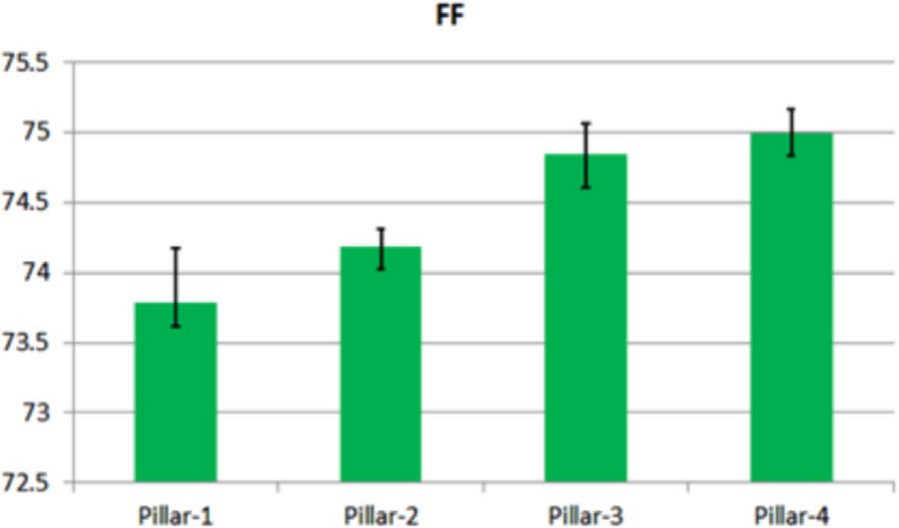
The variation of *FF*. Each bar represents the median value of each solar cell. The remarked line denotes the absolute error range.

**Fig. 4:**
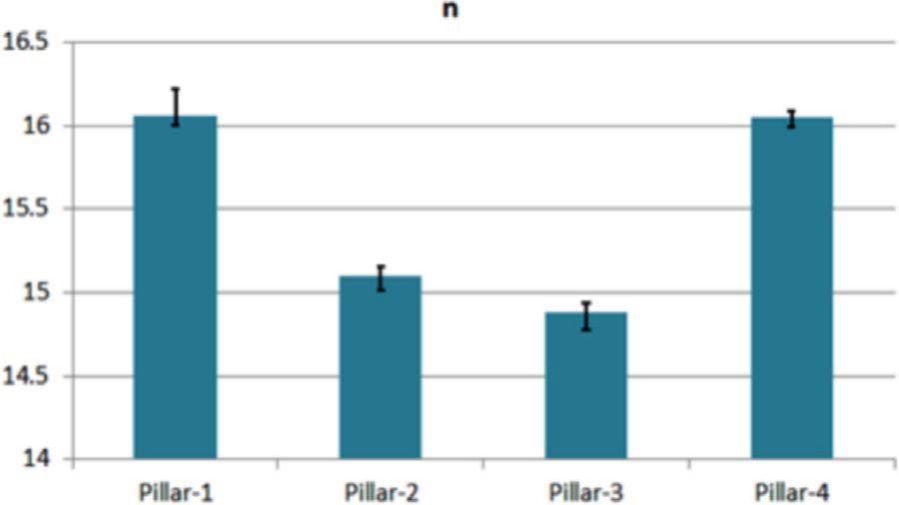
The variation of *n*. Each bar represents the median value of each solar cell. The remarked line denotes the absolute error range.

The datasets were acquired from the each SiMW solar cell. The repeating measurements (10 times) were performed on each solar cell to provide the accuracy of parameters. The measurement was obtained by an interval time of 1 min by changing of contact position. The accuracy of data is given for the open circuit voltage (*V_oc_*) for Fig. 1, short-circuit current (*J_sc_*) for Fig. 2, fill-factor (*FF*) values for Fig. 3, and overall efficiency (*n*) values for Fig. 4. The error bars are marked in each data figure. The overall accuracy of measurement is summarized in Table 1.

**Table 1:** Solar cell parameters with error bar distributed among the median values.

	*V_oc_* (mV)	*J_sc_* (mA/cm^2^)	*FF* (%)	*η* (%)
Pillar-1	606.65±1.25	35.871±0.256	73.785±0.383	16.06±0.16
Pillar-2	601.06±1.24	33.874±0.104	74.179±0.155	15.10±0.09
Pillar-3	587.64±0.46	33.823±0.115	74.848±0.241	14.88±0.10
Pillar-4	600.99±0.61	35.602±0.115	74.991±0.169	16.05±0.05
Flat-Si	549.36±0.84	24.511±0.127	73.611±0.182	9.90±0.07

## 2 Experimental design, materials and methods

### 2.1 Measurements

Four different shapes of microscale Si structures were fabricated for solar cells [1]. For the statistical analyses, each SiMW was measured by 10 times under one-sun illumination condition by using a solar simulator (K3000, McScience). The performances of solar cells (*V_oc_*, *J_sc_*, *FF*, and *n*) values were obtained by different contact position. Repeating of 10 times, the obtained data was calculated in terms of the median value and the absolute error. These statistical analyses may provide a scheme of the accuracy for solar cell performances.

Medianvalue=(Max.value–Min.value)/2Absoluteerror=Realvalue−Approximatevalue
